# Prostatic Inflammation Induces Fibrosis in a Mouse Model of Chronic Bacterial Infection

**DOI:** 10.1371/journal.pone.0100770

**Published:** 2014-06-20

**Authors:** Letitia Wong, Paul R. Hutson, Wade Bushman

**Affiliations:** 1 Department of Urology, University of Wisconsin-Madison, Madison, Wisconsin, United States of America; 2 Molecular and Environmental Toxicology Center, University of Wisconsin-Madison, Madison, Wisconsin, United States of America; 3 School of Pharmacy, University of Wisconsin-Madison, Madison, Wisconsin, United States of America; Northwestern University, United States of America

## Abstract

Inflammation of the prostate is strongly correlated with development of lower urinary tract symptoms and several studies have implicated prostatic fibrosis in the pathogenesis of bladder outlet obstruction. It has been postulated that inflammation induces prostatic fibrosis but this relationship has never been tested. Here, we characterized the fibrotic response to inflammation in a mouse model of chronic bacterial-induced prostatic inflammation. Transurethral instillation of the uropathogenic *E. coli* into C3H/HeOuJ male mice induced persistent prostatic inflammation followed by a significant increase in collagen deposition and hydroxyproline content. This fibrotic response to inflammation was accompanied with an increase in collagen synthesis determined by the incorporation of ^3^H-hydroxyproline and mRNA expression of several collagen remodeling-associated genes, including *Col1a1, Col1a2, Col3a1, Mmp2, Mmp9*, and *Lox*. Correlation analysis revealed a positive correlation of inflammation severity with collagen deposition and immunohistochemical staining revealed that CD45+VIM+ fibrocytes were abundant in inflamed prostates at the time point coinciding with increased collagen synthesis. Furthermore, flow cytometric analysis demonstrated an increased percentage of these CD45+VIM+ fibrocytes among collagen type I expressing cells. These data show–for the first time–that chronic prostatic inflammation induces collagen deposition and implicates fibrocytes in the fibrotic process.

## Introduction

Prostatic inflammation is a common finding in the adult prostate. It is more prevalent in men with benign prostatic hyperplasia (BPH) and lower urinary tract symptoms (LUTS) and the degree of prostatic inflammation correlates with severity and progression of symptoms [1], [2]. The mechanisms by which prostatic inflammation contributes to LUTS are a current focus of investigation. Several studies have suggested a correlation of prostatic fibrosis with increased urethral resistance and LUTS [3], [4]. Considering that prostatic inflammation and fibrosis are both associated with LUTS in men, it is postulated that prostatic inflammation contributes to the development and progression of BPH/LUTS by inducing prostatic fibrosis.

Inflammation has long been associated with fibrosis in other tissues but evidence for this in the prostate is only inferential. A recent study by Cantiello and colleagues, for example, showed that human prostate samples with evidence of inflammation had a significantly greater collagen content than those without inflammation [5]. However, a causal relationship between inflammation and fibrosis of the prostate has never been established. The purpose of this study was to examine directly whether inflammation causes fibrosis in a previously described mouse model of bacterial-induced prostatic inflammation [6], [7]. While there are significant species-specific differences in the anatomy, cellular and extracellular matrix compositions in the stroma of the mouse and human prostate [8], [9], the stromal-epithelial interactions are highly conserved between them [10] and the inflammatory responses to bacterial infection in the mouse prostate, in particular, appears to recapitulate the immunologic features of inflammation in the human prostate [7], [11]–[15]. We therefore expect our studies to provide insight into the causal relationship of inflammation and prostate fibrosis that are relevant to fibrosis of the human prostate.

Using a previously described model of bacterial-induced prostatic inflammation (6) we correlated prostatic inflammation with changes in collagen content using histological and biochemical quantitative methods at different time points after bacterial infection. We analyzed parameters related to inflammation and fibrosis including rates of collagen synthesis, the identity of collagen-producing cells, inflammatory infiltrate subtypes, and the changes in the mRNA expression of collagen remodeling-associated genes. Our observations validate the hypothesis that chronic inflammation induces prostatic fibrosis and describes, for the first time, the role of fibrocytes in the generation of prostatic fibrosis.

## Results

### Chronic Prostatic Inflammation Induced by Bacterial Infection

**Figure 1:**
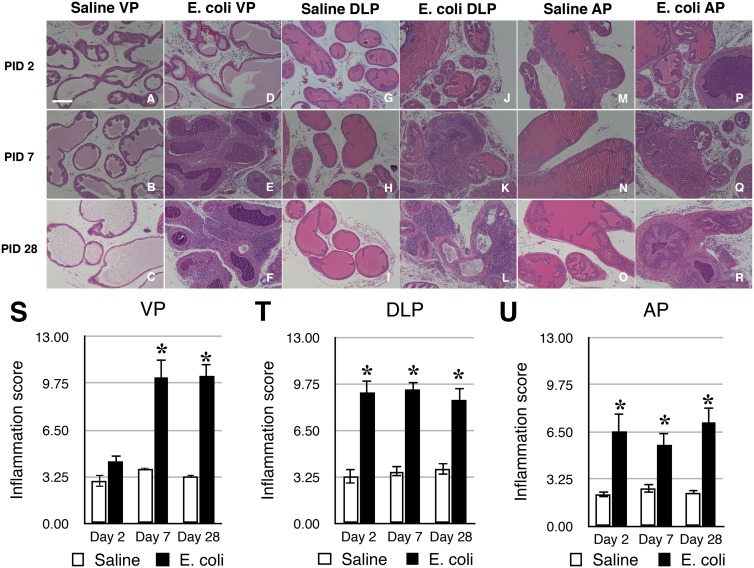
Prostatic inflammation induced by bacterial infection. **A–R.** Representative H&E stained sections of the VP (**A–F**), DLP (**G–L**), and AP (**M–R**) from saline instilled and *E. coli* infected C3H/HeOuJ male mice 2, 7, 28 days post-instillation. Scale bar 200 µm in panel A. **S–U.** Comparisons of the degree of inflammation in the VP (**S**), DLP (**T**) and AP (**U**) of saline instilled and *E. coli* infected C3H/HeOuJ male mice 2, 7, 28 days post-instillation (n = 4–13 per treatment per time point). Data are presented as the mean inflammation score ± SEM. *indicates a P-value<0.05 compared to saline control by two-sample t-test. Ventral prostate (VP); Dorsolateral prostate (DLP); Anterior prostate (AP).

Inbred C3H/HeOuJ male mice were instilled transurethrally with either sterile PBS or uropathogenic *E. coli 1677* as described previously [7]. This strain of mouse has previously been shown to remain infected and inflamed for at least 3 months post-inoculation [6]. Histopathological analysis was performed on the ventral prostate (VP), dorsolateral prostate (DLP), and anterior prostate (AP) of saline instilled and *E. coli* infected animals 2, 7, and 28 days post-inoculation. Saline instilled controls showed little or no inflammation whereas all lobes of the *E. coli* infected prostates displayed widespread mild-to-severe inflammation (Figure 1A–R). Significant inflammation was observed as early as on day 2 post-infection in the DLP and AP but not until day 7 in the VP. At 28 days post-inoculation, there was persistent inflammation in all three lobes (Figure 1S–U).

### Collagen Deposition in Response to Chronic Inflammation

**Figure 2:**
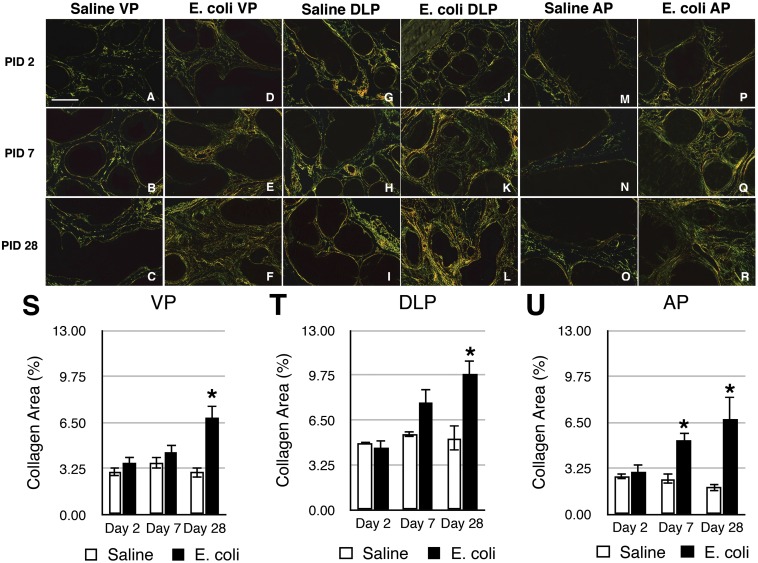
Collagen deposition is increased in response to chronic bacterial-induced prostatic inflammation. **A–R.** Representative picrosirius red stained sections of the VP (**A–F**), DLP (**G–L**), and AP (**M–R**) from saline instilled and *E. coli* infected C3H/HeOuJ male mice 2, 7, 28 days post-instillation. Scale bar 200 µm in panel A. **S-U.** Comparisons of the collagen content determined by the percentage of picrosirius red stained area in the VP (**S**), DLP (**T**) and AP (**U**) of saline instilled and *E. coli* infected C3H/HeOuJ male mice 2, 7, 28 days post-instillation (n = 4–13 per treatment per time point). Data are presented as the mean percentage of collagen area ± SEM. *indicates a P-value<0.05 compared to saline control by two-sample t-test. Ventral prostate (VP); Dorsolateral prostate (DLP); Anterior prostate (AP).

**Figure 3:**
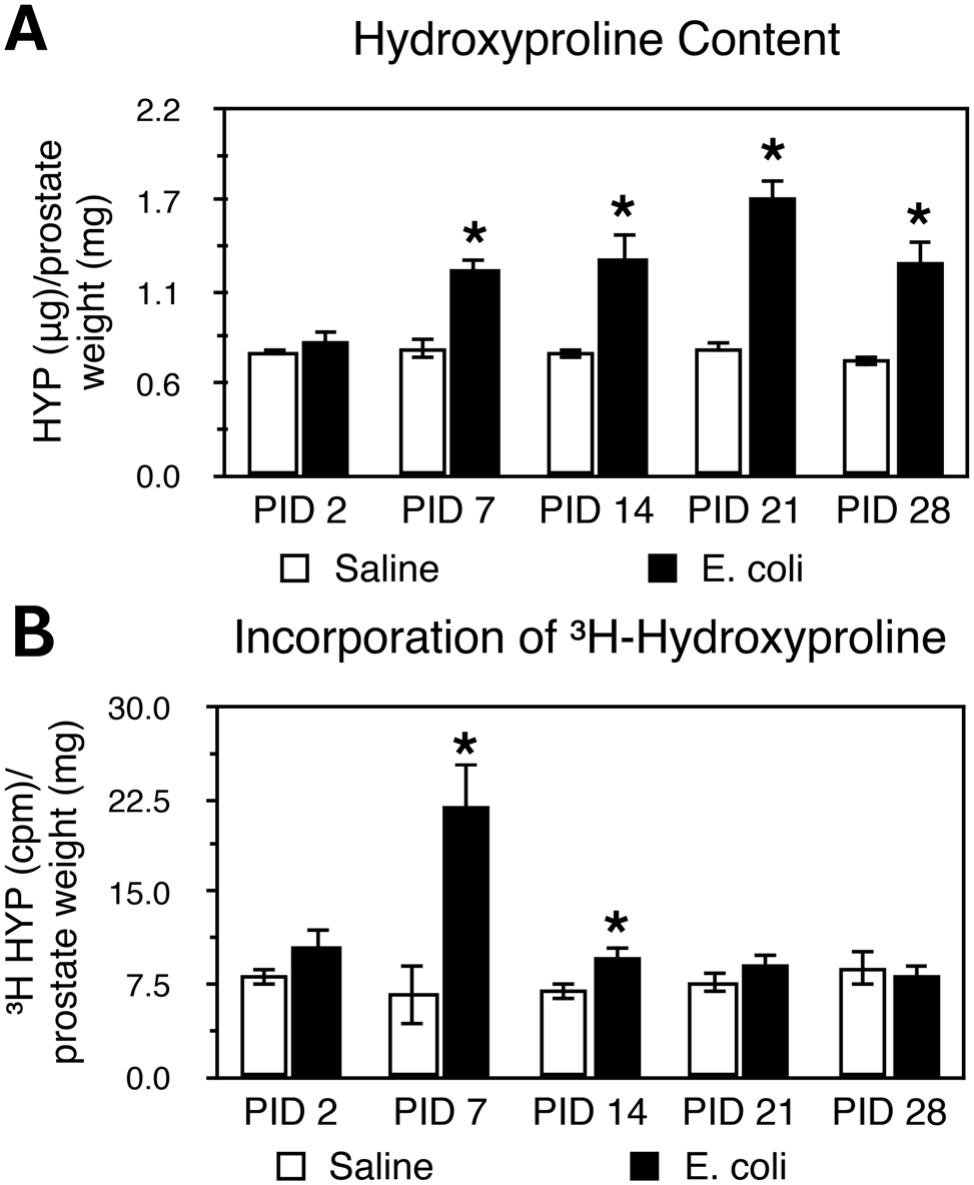
Hydroxyproline content and collagen synthesis are increased in bacterial-induced prostatic inflammation. **A.** Hydroxyproline content in saline instilled and *E. coli* infected prostates 2, 7, 14, 21, 28 days post-instillation (n = 4–8 per treatment per time point). Data are presented as mean hydroxyproline (µg)/prostate weight (mg) ± SEM. **B.** ^3^H-hydroxyproline incorporation in saline instilled and *E. coli* infected prostates 2, 7, 14, 21, 28 days post-instillation (n = 4–7 per treatment per time point). Data are presented as mean cpm/mg prostate ± SEM. *indicates a P-value<0.05 compared to saline control by two-sample t-test. Post-Instillation Day (PID); Hydroxyproline (HYP).

Picrosirius red staining for collagen content was performed on adjacent serial tissue sections of the H&E stained slides from saline instilled and *E. coli* infected male mice 2, 7, and 28 days post-instillation (Figure 2A–R). As shown in Figure 2S–U, quantitation of the staining showed no significant difference in collagen content of any lobe between saline instilled and *E. coli* infected animals on day 2 post-instillation. On day 7 post-instillation, a significant increase was observed in the AP of the infected mice. On day 28 post-instillation, all three prostatic lobes from infected mice showed a significant increase in collagen content. Increased collagen deposition on day 28 post-infection (Figure 2F, L, R) appeared to associate with the sites of inflammation as shown in the H&E images (Figure 1F, L, R). Hydroxyproline is a specific and unique amino acid found in collagen that can be used to measure collagen content. To validate the results obtained with picrosirius red staining, we quantitated hydroxyproline content by HPLC 2, 7, 14, 21, and 28 days post-instillation. Naïve animals and saline instilled animals 28 days post-instillation showed no significant difference in hydroxyproline content (Figure S1), confirming that instillation alone had no impact on collagen deposition. In agreement with the picrosirius red staining, no significant change in hydroxyproline content was observed 2 days post-instillation (Figure 3A). Hydroxyproline content was significantly increased on day 7 post-infection and remained increased until 28 days post-infection. We interpret our data as showing that chronic prostatic inflammation induces a significant and sustained increase in prostatic collagen content.

To determine if the increase in collagen content in chronic bacterial-induced prostatic inflammation is due to increased collagen synthesis, we measured the incorporation of ^3^H-hydroxyproline. Animals transurethrally either instilled with saline or infected with *E. coli* were i.p. injected with ^3^H-proline 0, 5, 12, 19, 26 days post-instillation and were sacrificed 48 hours later. ^3^H-hydroxyproline content was significantly increased in the infected prostates when assayed at 7 and 14 days post-infection as compared to saline controls. There was no significant difference when assayed at 21 and 28 days post-infection (Figure 3B). These findings reveal increased collagen synthesis during early phase of inflammation is responsible for the measured increase in collagen content associated with chronic inflammation.

### Correlation between Inflammation and Collagen Deposition

To evaluate the relationship between prostatic inflammation and collagen deposition, we correlated inflammation score determined from H&E images with collagen content determined from adjacent sections stained with picrosirius red for the VP, DLP, AP of saline instilled and *E. coli* infected animals 2, 7, and 28 days post-instillation (Table 1). On day 2 post-instillation, there was no correlation between the degree of inflammation and collagen content. At 7 days post-instillation the inflammation score was significantly and directly correlated with collagen content only in the AP. At 28 days post-instillation all three prostatic lobes showed a significant positive correlation between inflammation and collagen content.

**Table 1:** Correlation between inflammation and collagen deposition in the VP, DLP, and AP from saline instilled and *E. coli* infected mice (n = 4–13 per treatment per time point).

	VP		DLP		AP	
	ρ	P-value	ρ	P-value	ρ	P-value
**PID 2**	0.54	0.11	−0.26	0.47	0.13	0.72
**PID 7**	0.06	0.86	0.30	0.43	0.89	0.0005
**PID 28**	0.65	0.0013	0.58	0.0056	0.71	0.0004

ρ: Spearman’s rank correlation coefficient; VP: Ventral prostate; DLP: Dorsolateral prostate, AP: anterior prostate.

### Collagen Remodeling-associated Gene Expression in the Inflamed Prostate

**Figure 4:**
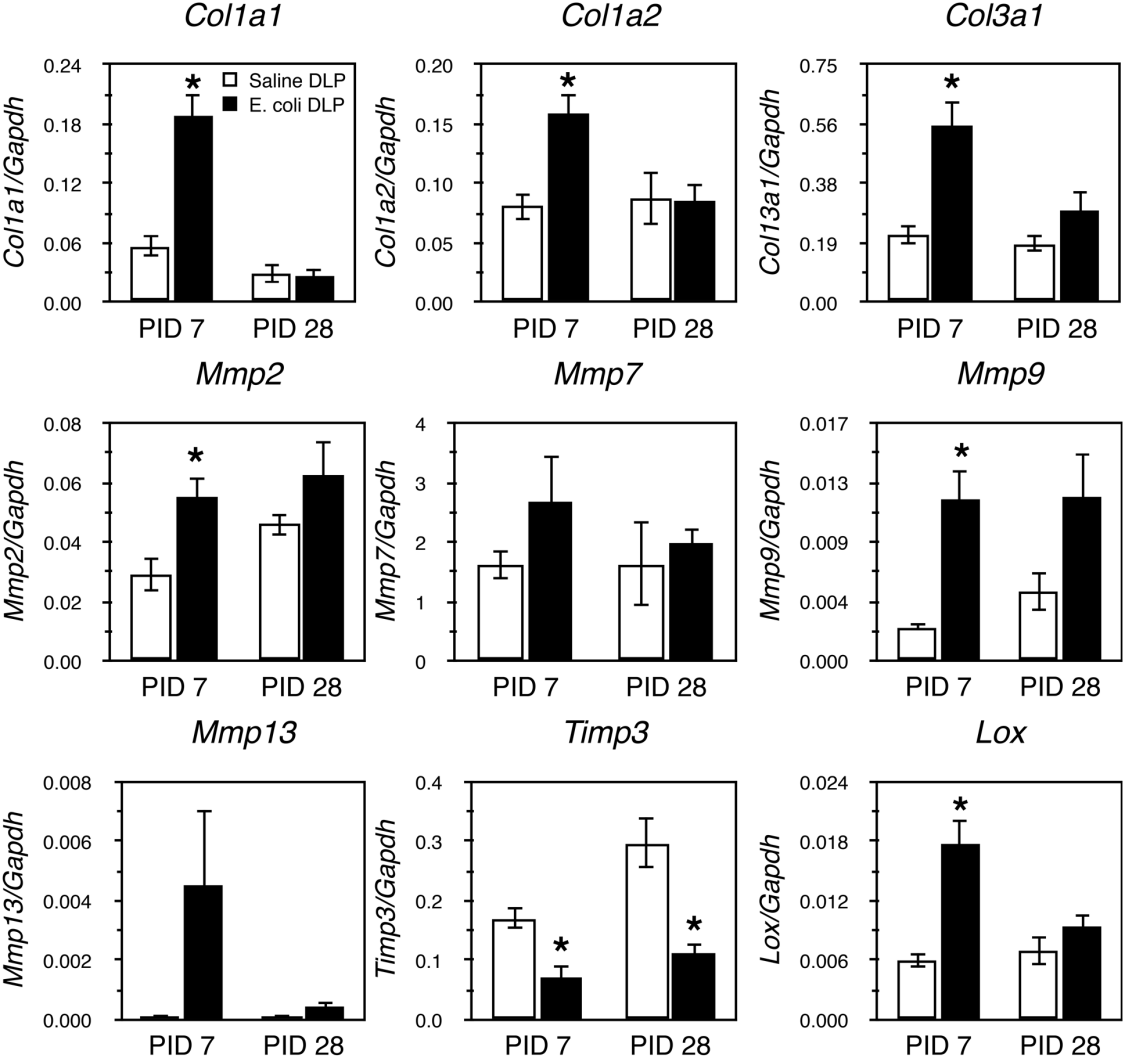
Changes in collagen remodeling-associated gene expressions during bacterial-induced prostatic inflammation. qRT-PCR for collagen remodeling-associated genes in the DLP of saline instilled and *E. coli* infected animals 7 and 28 days post-instillation (n = 4–7 per treatment per time point). Data are presented as mean gene expression ± SEM. Gene expression levels were normalized to the housekeeping gene *Gapdh.* *indicates a P-value<0.05 compared to saline control by two-sample t-test. Post-Instillation Day (PID); Dorsolateral prostate (DLP).

We analyzed mRNA expression pattern of collagen remodeling-associated genes in the DLP (Figure 4) of saline instilled and *E. coli* infected mice 7 and 28 days post-instillation. Robust changes in expression of several genes was evident 7 days post-infection; expression of *Col1a1, Col1a2, Col3a1, Mmp2, Mmp9* and *Lox* was significantly increased while expression of *Timp3* was significantly decreased. No significant changes were observed in the expression of *Col4a1, Col6a1, Col6a2, Mmp7 and Mmp13* (Figure 4, Figure S2). In striking contrast, we observed no differences in expression of any collagen remodeling-associated genes except *Timp3* 28 days post-instillation. Thus, the time course of increased expression of collagen remodeling-associated genes seems to parallel active collagen synthesis as determined by measurement of ^3^H-hydroxyproline incorporation.

### Characterization of Inflammatory Infiltrates

**Figure 5:**
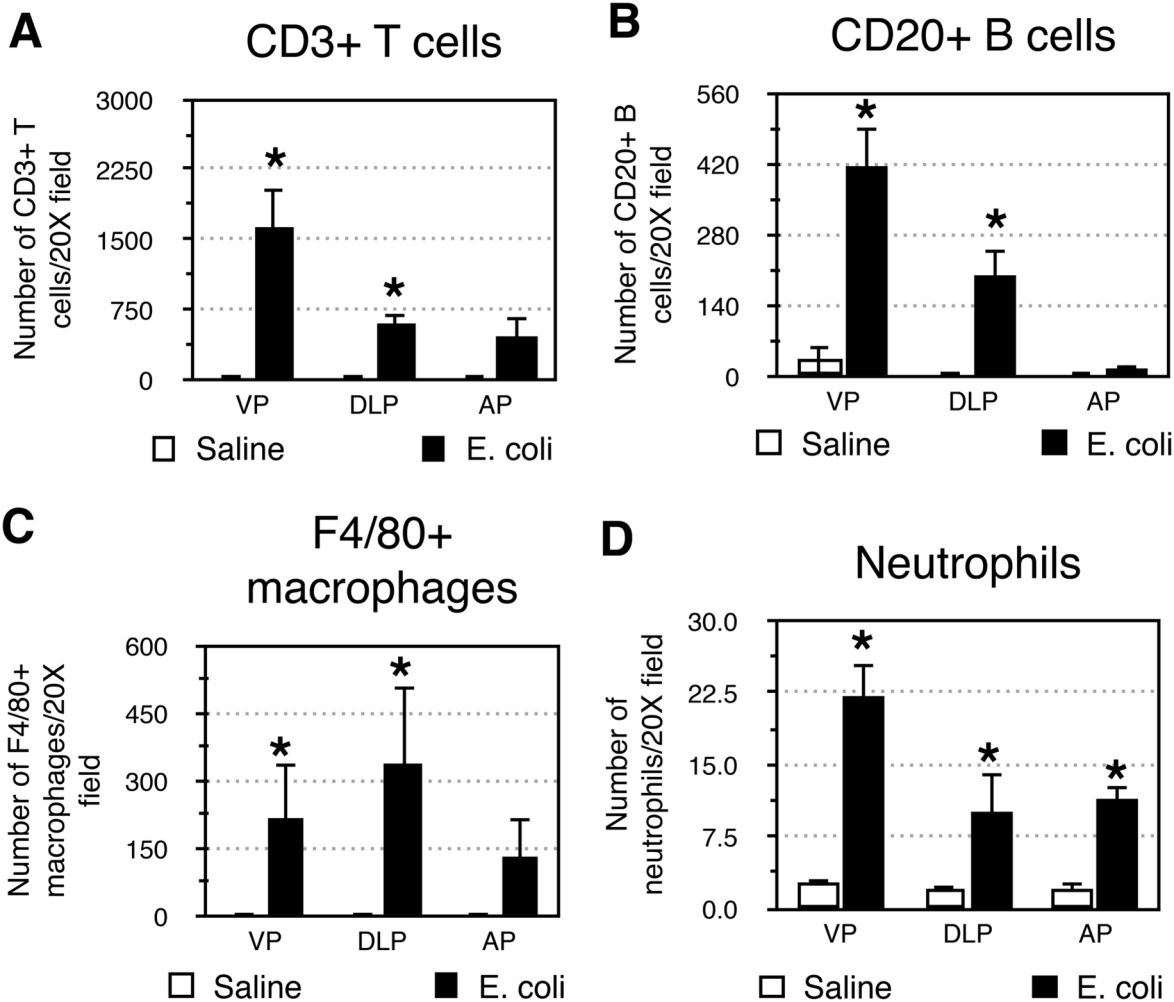
Characterization of inflammatory infiltrate in the inflamed prostate. Comparisons of the number of CD3+ T cells (**A**), CD20+ B cells (**B**), F4/80+ macrophages (**C**), and neutrophils (**D**) in the VP, DLP, and AP from saline instilled and *E. coli* infected mice 28 days post-instillation. n = 4 per group. Data are presented as the mean number of cells per 20X field. *indicates a P-value<0.05 compared to saline control by Wilcoxon rank-sum test. Ventral prostate (VP); Dorsolateral prostate (DLP); Anterior prostate (AP).

**Figure 6:**
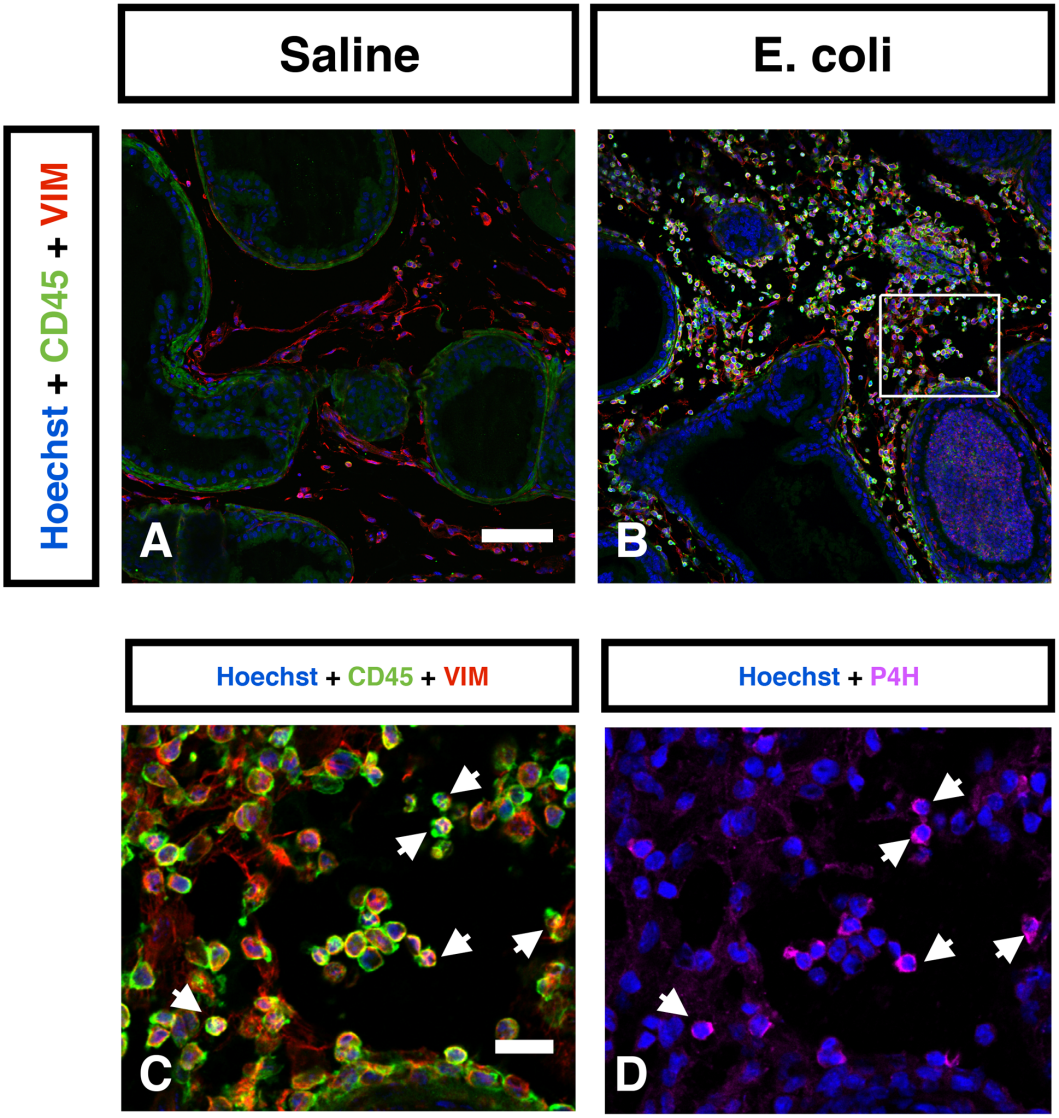
Identification of collagen-producing cells in the inflamed prostate. Representative IHC images of CD45 (Green) and vimentin (Red) in the saline instilled (**A**) and *E. coli* infected (**B**) prostates 7 days post-instillation. n = 4–6 per group. Hoechst nuclear staining is shown in blue. Scale bar 100 µm in panel A. **C.** High magnification inset (box in panel B) shows abundant number of CD45+VIM+ fibrocytes in the *E. coli* infected prostate. Scale bar 20 µm in panel C. **D.** Immunostaining for prolyl 4-hydroxylase (Magneta) in the same field as panel C. White arrows indicate localization of prolyl 4-hydroxylase to CD45+VIM+ fibrocytes. Vimentin (VIM); prolyl 4-hydroxylase (P4H).

**Figure 7:**
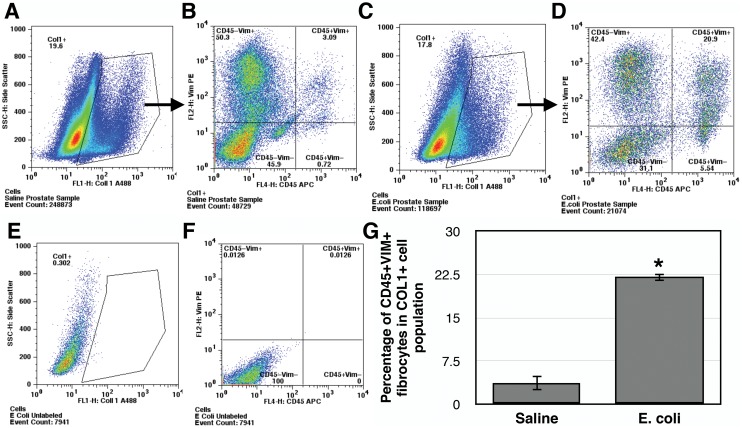
CD45+VIM+ fibrocytes are enriched in the population of COL1+ cells in the inflamed prostate. Representative flow cytometric analyses of freshly isolated prostatic cells from saline instilled (**A–B**) and *E. coli* infected (**C–D**) prostates 7 days post-instillation triple stained for the expression of COL1, CD45 and vimentin. The cells were gated for COL1 expression (A and C) and subsequently COL1 positive cells were analyzed for CD45 and vimentin expression (B and D) based on unstained controls. Representative flow cytometric analyses of unstained prostatic cells control with gate parameter for COL1 were shown in **E** and vimentin/CD45 in **F**. **G.** Comparison of the percentage of CD45+VIM+ fibrocytes in COL1 expressing cell population from the prostates of saline instilled and *E. coli* infected mice. Data are presented as mean percentage ± SEM. Prostatic tissue cells were pooled from 4–6 mice per group and total 3 independent experiments were performed. *indicates a P-value<0.05 compared to saline control by two-sample t-test.

The inflammatory infiltrates present in the VP, DLP, and AP were characterized 28 days after bacterial inoculation. CD3+ T cells (Figure 5A), CD20+ B cells (Figure 5B) and F4/80+ macrophages (Figure 5C) were the predominant inflammatory cells found in all three *E. coli* infected prostatic lobes. A relatively small but significant number of neutrophils were also present (Figure 5D). To determine the cell type responsible for collagen synthesis associated with inflammation we performed immunohistochemical costaining 7 days post-instillation, a time point that corresponds to the peak of ^3^H-hydroxyproline incorporation in the inflamed prostate. Staining for vimentin and α-smooth muscle actin surprisingly showed no evidence of VIM+αSMA+ myofibroblasts in either the saline instilled or *E. coli* infected prostates (Figure S3). In contrast, staining for vimentin and CD45 showed abundant number of CD45+VIM+ fibrocytes in the *E. coli* infected prostates while only minimal number of fibrocytes was detected in the saline instilled prostates (Figure 6A–B). To determine if these fibrocytes are involved in collagen synthesis we performed staining for prolyl 4-hydroxylase (P4H), a key enzyme involved in collagen synthesis that catalyzes the conversion of proline to hydroxyproline before collagen secretion. Triple immunohistochemical staining for vimentin, CD45 and P4H in the *E. coli* infected prostates demonstrated P4H expression in a subpopulation of CD45+VIM+ fibrocytes (Figure 6C–D). Further assessment of prostatic cells isolated from saline instilled and *E. coli* infected mice using flow cytometry demonstrated that CD45+VIM+ fibrocytes are present within collagen type I (COL1) positive cells. Consistent with our immunohistochemical finding, there was a 6-fold increase in the percentage of CD45+VIM+ fibrocytes enriched in COL1 expressing cell population from the *E. coli* infected prostates (Figure 7). This observation strongly suggests fibrocytes as collagen-producing cells in chronic bacterial-induced prostatic inflammation.

## Discussion

The collagens are a major group of extracellular matrix proteins. To date, 28 types of collagen have been identified in vertebrates and are grouped into subclasses based on their structural characteristics. The majority of collagen subtypes form fibrils. Others are network-forming collagens, transmembrane collagens or collagens associated with fibrils [16]. As a group, they play an important role in maintaining structural integrity of various tissues and organs. They are best known to provide tensile strength to the tissues but also have significant roles in tissue scaffolding, cell migration, cell adhesion, morphogenesis and tissue repair [17]–[19]. The collagen content of the extracellular matrix of the tissues is a function of collagen synthesis and degradation. Dysregulation of collagen turnover resulting in excess collagen accumulation (fibrosis) can disrupt organ structure and function [20]–[23] and fibrosis is associated with the development and progression of disease in many different organs, including the lung, liver, intestine and heart [24]–[31].

Collagen remodeling and deposition is cardinal feature of the response to injury and is necessary to restore mechanical strength and function of the injured tissue [17], [32]. Immune regulation is critical to prevent excess collagen deposition [33]–[35]. When inflammation fails to resolve – typically because of persistent tissue injury – a pathologic wound healing response occurs that can produce fibrosis. Fibrosis is generally characterized by an accumulation of fibrogenic cells and excessive deposition of extracellular matrix components [36]. A mouse model of bleomycin-induced lung injury and inflammation showed that repeated injury was accompanied by a loss of normal tissue architecture, a persistent increase in collagen deposition, increased collagen synthesis and increased number of myofibroblasts [37]. The fibrotic change in lung inflammation was associated with reduced total lung capacity, increased dynamic resistance and decreased dynamic compliance [38]. Subsequent studies showed that a variety of inflammatory cytokines and chemokines including TGFβ, CXCL6, IL-1, IL-5, IL-6, IL-17 modulate collagen deposition and the fibrotic effects of lung inflammation [39]–[44].

We observed significant collagen deposition with chronic inflammation induced by bacterial infection. Inflammation present on day 2 post-inoculation is characterized by an acute inflammatory infiltrate composed of polymorphonuclear cells and macrophages [7]. At later time points, the inflammation is characterized by a chronic inflammatory infiltrate composed predominantly of T cells, B cells and macrophages. Our quantitative studies revealed that collagen deposition occurs primarily in the early stage of chronic inflammation. Collagen accumulation was associated with the sites of inflammation and we observed a significant positive correlation between collagen content and the severity of inflammation. The observed spatial and temporal association between inflammation and collagen accumulation supports the concept that excessive collagen deposition is a response to chronic inflammation.

We found that collagen deposition in chronic bacterial prostatic inflammation was associated with an increase in *de novo* collagen synthesis and collagen remodeling-associated gene expression early in the chronic inflammatory phase. Incorporation of ^3^H-hydroxyproline was significantly increased at 7 and 14 days post-inoculation and then decreased to baseline. Elevated collagen synthesis was accompanied by increased expression of collagens (*Col1a1, Col1a2, Col3a1*), crosslinking enzyme lysyl oxidase (*Lox*), and matrix metalloproteinases (*Mmp2, Mmp9*) and decreased expression of the tissue inhibitor of metalloproteinases (*Timp3*). These factors coordinately regulate the processes of collagen synthesis, degradation and stabilization. Collagen I and III are the predominant fibrillar types of collagens upregulated in fibrotic conditions associated with inflammation. The increase in expression of these collagen subtypes in our study occurred in parallel with increased collagen deposition, suggesting that collagen synthesis, partly due to enhanced transcriptional level, contributes to collagen accumulation in persistent prostatic inflammation. In addition to increased collagen synthesis, stabilization of collagen fibrillar structures through formation of collagen cross-links by LOXs renders collagen to be less susceptible to degradation mediated by collagenases and therefore could further contribute to its accumulation [45]. Conversely, MMPs, whose proteolytic activity is inhibited by TIMPs, cleave and remodel extracellular matrix components as well as release and activate proinflammatory and profibrotic cytokines, constituting a microenvironment favoring fibrogenic process [46].

Fibroblasts/myofibroblasts are traditionally considered as the main sources of extracellular matrix components in tissue repair and inflammation [47]. However, there is a growing body of research supporting the involvement of a unique population of bone marrow derived inflammatory fibroblast-like cells called fibrocytes in the development of fibrosis in chronic inflammatory diseases, including pulmonary fibrosis [48]–[52], autoimmune disorders [53], [54], asthma [55], [56], and chronic kidney disease [57], [58]. Fibrocytes are collagen-producing cells that have been classically identified based on their distinctive expression of the common leukocytic surface marker CD45, the hematopoietic stem cell marker CD34 and the fibroblastic markers collagen I, vimentin, and/or prolyl 4-hydroxylase [59], [60]. An increased number of fibrocytes has been reported in the circulation and the lungs of patients with fibrotic interstitial lung disease as compared to that in healthy subjects [50]–[52]. In the mouse model of bleomycin-induced pulmonary fibrosis, the intrapulmonary recruitment of fibrocytes was dramatically increased and correlated with increased collagen deposition in the lungs [49]. Inhibition of fibrocyte infiltration to the lungs of bleomycin-treated mice significantly reduced lung fibrosis, suggesting that fibrocytes could directly contribute to the pathogenesis of fibrosis.

Our data suggests that fibrocytes but not myofibroblasts are a key feature of the fibrotic response to bacterial-induced prostatic inflammation. Whereas immunostaining for vimentin and αSMA showed no evidence of VIM+αSMA+ myofibroblasts in the infected prostates, immunostaining for CD45, vimentin, and prolyl 4-hydroxylase revealed that the *E. coli* infected prostates had abundant CD45+VIM+ fibrocytes of which a subpopulation also expressed prolyl 4-hydroxylase, a key enzyme essential for collagen synthesis. Flow cytometric analysis further demonstrated that CD45+VIM+ fibrocytes are a subpopulation of collagen type I expressing cells in the prostates and there was a significant increase in the percentage of CD45+VIM+ fibrocytes in COL1+ cells post-infection. The appearance of these CD45+VIM+ fibrocytes in the prostates following 7 days of bacterial infection was temporally associated with enhanced collagen synthesis and increased collagen deposition and suggests, therefore, that CD45+VIM+ fibrocytes have a significant if not primary role in prostatic fibrosis. It is noteworthy that a recent study documented the presence of a cell population phenotypically consistent with fibrocytes in the prostate of human patients with LUTS [61]. However, these authors did not characterize the role of this specific cell population in BPH/LUTS. Therefore, to our knowledge, the present study is the first report in the literature to describe fibrocytes in prostatic inflammation and fibrosis.

There is accumulating evidence for the contribution of increased collagen deposition and fibrosis to the pathogenesis of BPH/LUTS [3]–[5], [62]; however, the underlying cause of increased collagen deposition in this common aging-related disease has not been elucidated. Experimental studies have observed a potential link between inflammation and extracellular matrix alterations in the prostate in both *in vitro* and *in vivo* studies. Barron and colleagues have shown that a transgenic mouse model constructed with constitutively active *Tgfb1* in prostate epithelium exhibited an age-dependent inflammation concomitant with the increase in collagen deposition in the prostates [63]. Another study by Gharaee-Kermani, et al. has demonstrated that treatment of primary human prostate stromal fibroblasts isolated from BPH patients with inflammatory factors such as TGFβ1, CXCL5, CXCL8, CXCL12 induced their collagen expression [61]. Recently, there are several retrospective studies of BPH/LUTS specimens reporting a positive relationship between the degree of chronic inflammation and prostatic fibrosis [4], [5]. Although these studies suggested that prostatic inflammation might play a role in promoting the fibrotic changes in BPH/LUTS, this has never been directly verified. Our results support this hypothesis that prostatic chronic inflammation induces collagen deposition and, for the first time, suggest a key role for fibrocytes in inflammation-associated prostatic fibrosis.

## Materials and Methods

### Study Approval

This study was carried out in strict accordance with the recommendations in the Guide for the Care and Use of Laboratory Animals of the National Institutes of Health. All animal studies were approved by the Institutional Animal Care and Use Committee at the University of Wisconsin-Madison (M02448). All surgery was performed under isoflurane anesthesia and all sacrifice was performed under isoflurane anesthesia immediately followed by cervical dislocation. All efforts were made to minimize animal suffering.

### Transurethral Instillation

Transurethral instillation was performed as previously described [7]. 8 week old C3H/HeOuJ male mice (Jackson Laboratories) were anesthetized with isoflurane and catheterized with a lubricated sterile polyethylene catheter per urethra. Inoculation was performed by a single transurethral instillation of uropathogenic *E. coli* 1677 (2×10^6^ CFU/ml) or sterile PBS in a volume of 200 µl.

Animals were sacrificed 2, 7, 14, 21, and 28 days post-instillation. The paired prostatic lobes (VP, DLP, AP) were bisected separately and were used for RNA isolation, hydroxyproline assay, and/or histology. The whole mouse prostates were collected and used for measuring incorporation of ^3^H-hydroxyproline to determine collagen synthesis. Four to thirteen mice per time point and per treatment were analyzed for histology. Four to seven mice per time point and per treatment were used for gene expression analysis. Four to eight mice per time point and per treatment were used for hydroxyproline assay. Four to seven mice per time point and per treatment were used for determination of collagen synthesis. Four to six mice per treatment were used for FACS analysis.

### Quantitation of Inflammatory Degree

#### Inflammatory grading

Tissues were fixed in 10% formalin, imbedded in paraffin and serially sectioned at 6 µm. Standard H&E staining was performed for histology. Using our previously established scoring system [7], the severity of inflammation was graded in at least 3 random 10X fields of H&E sections from each prostatic lobe. Data are presented as the mean inflammation score ± standard error of the mean (SEM).

#### Characterization of inflammatory infiltrates

Neutrophils were identified as polymorphonuclear leukocytes and counted on 20X fields of H&E stained slides. T-lymphocytes, B-lymphocytes, and macrophages were counted by using immunohistochemistry for specific cell surface markers as previously described [7]. 6 µm formalin-fixed paraffin-embedded sections were used. Antigen retrieval was performed by boiling in citrate buffer solution (pH 6.0) for 10 minutes and blocking for nonspecific background was achieved by using 10% donkey serum and 1% BSA in PBS for 1 hour at room temperature. Slides were incubated with primary antibodies in blocking buffer at 4°C overnight and secondary antibodies for one hour at room temperature in dark. Sections were counterstained with Hoechst 33258 at 4 µg/ml in PBS, coverslipped with anti-fade media and imaged. Primary antibodies included rabbit polyclonal anti-CD3 (1∶100, DAKO A0452), goat polyclonal anti-CD20 (1∶50, Santa cruz sc-7735), and rat monoclonal anti-F4/80 (1∶50, eBioscience 14-4801-85). Secondary antibodies included donkey anti-rabbit IgG-Alexa 594 conjugated antibody (1∶200, Invitrogen), donkey anti-goat IgG-Alexa 488 conjugated antibody (1∶100, Invitrogen), and donkey anti-rat IgG-Alexa 594 conjugated antibody (1∶50, Invitrogen), respectively. Data are presented as the mean number of cells ± SEM.

### Quantitation of Collagen Content

#### Picrosirius red staining

Adjacent serial tissues sections of H&E stained slides were used for picrosirius red staining. The staining was performed by incubating slides in 0.1% sirius red in saturated aqueous solution of picric acid for one hour at room temperature. Images were taken by digital camera using NIS Element software under a Nikon Eclipse 80i polarized light microscope. The settings of the software and microscope remained unchanged throughout the observation for the purpose of quantitation and comparisons. The color staining that shows up under polarizing microscope corresponds to the birefringence of collagen fibers [64]. Quantitation of the staining for collagen content was then determined by using NIH Image J. Briefly, the images were converted from RBG to 8-bit gray scale and the intensity of the three color channels was summed into one image. The images were manually thresholded to define the collagen staining and a constant value of the threshold was set for all the images analyzed. The region of interest (prostatic tissues) was manually outlined to analyze the area that was stained for collagen. Large blood vessels and nerve fiber bundles in the prostate were eliminated in the quantitation. Data are presented as the mean percentage of collagen area ± SEM.

#### Hydroxyproline assay for collagen content by HPLC

Mouse prostate tissues were harvested, snap frozen in liquid nitrogen, and stored at −80°C until further analysis. The procedure was performed as previously described with some modifications [65]. The tissues were thawed on ice and weighed. Each tissue sample was homogenized in 2 ml of 12N HCl with a motorized homogenizer in a clean glass tube. 75 µl of 20 mM sacrosine (reagent grade) as internal standard was mixed in the homogenate. Then, the glass tubes containing the tissue homogenate were tightly capped to prevent evaporation and were incubated in a 110°C heating block for 18 hours. The hydrolysates were allowed to cool to room temperature, neutralized with 2 ml of 12N NaOH and 1 ml of borate buffer (0.7 M boric acid in water, pH 9.5 with NaOH), and were adjusted to a pH 9.5 with NaOH and HCl. Aliquots of 500 µl of the sample solution were used for subsequent derivatization process. Some samples were run in duplicate or triplicate for intra-day reaction consistency and these same samples were run on the next day for inter-day reaction consistency. Hydroxyproline (reagent grade) standards of 0.3125, 0.625, 1.25, 2.5, 5, 10, 20 mM with 7.5 mM L-proline (reagent grade) in water were prepared. 200 µl of each hydroxyproline standard was mixed with 75 µl of 20 mM sacrosine, 1.8 ml of 12N HCl, 2 ml of 12N NaOH and 1 ml of borate buffer. The solutions were then adjusted to pH 9.5. Aliquots of 500 µl of the hydroxyproline standard solution were used for subsequent derivatization process.

Aliquots (500 µl) of tissue homogenate or hydroxyproline standard were added with 700 µl of borate buffer. The following procedures were performed in dark. The solutions were mixed with 100 µl of OPA solution (50 mg *o*-phthalaldehyde dissolved in 1 ml acetonitrile and 26 µl of reagent grade β-mercaptoethanol) by vortexing and allowed to react at room temperature for 1 minute. The solution was then mixed with 100 µl of iodoacetamide solution (140 mg/ml of iodoacetamide in acetonitrile) by vortexing and reacted at room temperature for 1 minute. 600 µl of 5 mM FMOC-Cl in acetone was subsequently added, mixed by vortexing and reacted at room temperature for 1 minute. The solutions were then washed three times with 3 ml of ethyl ether by vortexing for 30 seconds. The organic layer was discarded each time. 50 µl of the aqueous phase was injected into the HPLC. A total of 3 injections were run for each reacted sample or standard. Sample injections were made every 25 minutes without an intervening wash. The isocratic mobile phase was prepared by combining 650 ml of 3% glacial acetic acid that was adjusted to pH 4.3 with sodium acetate (reagent grade) with 350 ml of acetonitrile, followed by vacuum filtration for degassing. All reagents used were ACS or HPLC grade unless stated otherwise.

The HPLC instrumentation and spectrofluorometer was set up as previously described [65]. The HPLC instrumentation included a Shimadzu LC-10AD HPLC pump, SIL-10A auto injector and system controller. A McPherson Model FL-750 spectrofluorometer was used with the high-sensitivity module, an excitation wavelength of 265 nm and without an emission cut-off filter. Separation was achieved by using a Lichrosphere 5 RP18e 250 mm×4.60 mm, 5 µm column. The mobile phase was pumped at a constant rate of 0.75 ml/min.

The coefficients of variation for intra-day reaction consistency were less than 4.5% and for inter-day reaction consistency were less than 10.2%. The height ratio of the internal standard (sarcosine) and hydroxyproline peak was calculated for each sample and standard. The exact amount of hydroxyproline standards in µg injected into the HPLC was determined by calculating the total dilution made from the original concentration prepared. The standard curve of hydroxproline standards showed linear regression with R^2^ = 0.998. The amount of hydroxyproline in µg presented in the prostate samples was calculated from the peak height ratio of hydroxyproline and internal standard peak into the linear regression equation obtained from the hydroxyproline standard curve. Data are presented as mean hydroxyproline (µg)/prostate weight (mg) ± SEM.

### Collagen Synthesis by Incorporation of ^3^H-hydroxyproline

The animals instilled transurethrally with either sterile PBS or uropathogenic *E. coli* 1677 were i.p. injected with 15 µCi of L-[3,4-^3^H]-proline (specific activity 50 Ci/mmol, ARC, Inc.) on day 0, 5, 12, 19, 26 post-instillation. For day 0 post-instillation, animals were injected 2–3 hours after bacterial inoculation. Prostates were harvested 48 hours after ^3^H-proline injection, snap frozen in liquid nitrogen, and stored at −80°C until further analysis. The tissue samples were thawed on ice and weighed. The extraction procedure of hydroxyproline has previously been described in detail [66]. Briefly, each tissue sample was placed in a clean glass tube, homogenized in 2 ml 12N HCl with a motorized homogenizer and was heated at 110°C for 18 hours. The hydrolysates were then allowed to cool to room temperature and neutralized with 2 ml of 12N KOH and 1 ml of 1 M boric acid in water. The samples were then adjusted to pH 8.7 with KOH and HCl. Aliquots of 2 ml of the sample solution were used for subsequent reaction. First, each sample was reacted with 4 ml of 0.2 M chloramine T-solution in methoxyethanol at room temperature for 20 minutes. 2.4 ml of 3.6 M sodium thiosulfate in water was then added followed by saturation with 1.5 g KCl. The solutions were then washed 3X with 9 ml of toluene by vigorously shaking for 5 minutes each. Organic layer was discarded each time. The samples were heated in boiling water for 30 minutes and then allowed to cool down on ice for 10 minutes. Then, 7 ml of toluene was added into each sample and mixed by vigorously shaking for 10 minutes. The organic layer that contained hydroxyproline was then removed and mixed with 7 ml of liquid scintillation cocktail (Fisher, SX25-5) in a 20 ml liquid scintillation vial. Counts per minute (cpm) were measured with a Beckman LS 6000TA liquid scintillation counter. Data are presented as mean ^3^H-hydroxyproline cpm/mg prostate ± SEM.

### Immunohistochemical Staining

Immunohistochemical costaining for vimentin, CD45 and P4H was used to assess the presence of fibrocyte in the prostate and the expression of prolyl 4-hydroxylase in fibrocyte. 6 µm sections of formalin-fixed paraffin-embedded tissues were dewaxed and rehydrated by passing them through xylene 3X for 5 minutes each, 100% methanol 3X for 5 minutes each, and then running tap water for 10 minutes. The slides were submerged in boiling citrate buffer solution (pH 6.0) for 10 minutes for antigen retrieval, and blocked for nonspecific background by using 10% donkey serum and 1% BSA in PBS for 1 hour at room temperature. Slides were then washed three times with PBS+0.025% Tween 20 (PBST) for 5 minutes. Primary antibodies including rabbit monoclonal anti-vimentin (1∶100, Abcam ab92547), rat monoclonal anti-CD45 (1∶50, Abcam ab25386), and goat polyclonal anti-P4H (1∶50, Abcam ab59497) were added in the blocking solution. The slides were incubated with primary antibodies at 4°C overnight followed by three washes with PBST for 5 minutes each. The slides were then incubated with the secondary antibodies in blocking solution for 1 hour at room temperature in the dark. Secondary antibodies included donkey anti-rabbit IgG-Alexa 594 conjugated antibody (1∶100, Invitrogen), donkey anti-rat-Alexa 488 conjugated antibody (1∶100, Invitrogen), and donkey anti-goat-Alexa 647 conjugated antibody (1∶200, Invitrogen). Following incubation with secondary antibodies the slides were rinsed three times with PBST for 5 minutes. Nuclei staining was performed by incubating slides with Hoechst 33258 at 4 µg/ml in PBST at room temperature for 10 minutes. The slides were then washed 3X with PBST and 2X with distilled water for 5 minutes each. The slides were mounted with anti-fade media, a coverslip and imaged. Images were taken by a digital camera using NIS Element software under Nikon Eclipse Ti-E fluorescent microscope.

### Fluorescence Activated Cell Sorting (FACS) Analysis for Collagen Producing Fibrocytes

Prostatic tissues were minced, digested with 1X collagenase/hyaluronidase at 37°C for 3 hours, 0.25% trypsin on ice for 1 hour and 5 U/ml dispase at room temperature for 2 minutes. Cells were passed through a 22-gauge needle and then a 100-µm cell strainer to produce a single cell suspension. Cells were resuspended into 100 µl 2% FBS/DMEM/F12, incubated with mouse Fc Block (1 µg/sample, BD Pharmingen #553142) at 4°C for 15 minutes and washed. Each sample was then divided into 5 aliquots: (a) unstained control, (b) CD45 only control, (c) Vimentin only control, (d) collagen type I only control, and (e) CD45, Vimentin, collagen type I sample. Cells were stained with APC labeled rat anti-mouse CD45 (1∶20, BD Pharmingen #559864) at 4°C for 30 minutes in dark, washed, fixed and permeabilized with BD Cytofix/Cytoperm (BD Pharmingen #554714) at 4°C for 20 minutes. Cells were then stained with PE labeled rabbit anti-mouse Vimentin (1∶20, Cell Signaling Technology #12020) and rabbit anti-mouse collagen type I (1∶100, Rockland Immunochemical) conjugated with Alexa Fluor 488 using Zenon Rabbit IgG Labeling Kits (Invitrogen #Z-25302) at 4°C for 30 minutes in dark and washed. A three-color analysis of stained cells was performed on a flow cytometer (BD Biosciences FACSCalibur) using Cellquest software and data analysis was subsequently performed using FlowJo software. Unstained and single antibody-stained cells were used as controls. The gate parameters were set based on the findings in controls.

### RNA Isolation and Semi-quantitative Real-time PCR

RNA from the tissues was extracted using RNeasy Micro Kit (Qiagen, Inc.) and converted to cDNA as previously described [7]. Semi-quantitative RT-PCR was performed with cDNA samples to quantitate gene expression levels. The forward and reverse primers of *Col1a1, Col1a2, Col3a1, Mmp2, Mmp7, Mmp9, Mmp13, Timp3,* and *Lox* were designed using the NCBI mouse nucleotide database, the mouse genomic BLAST database and the Primer-3 program. The sequences of the primers used are shown in Table S1. RT-PCR cycle reactions were detected with SYBR green (Roche) and run on a BioRad Real-Time CFX with run conditions of 95°C for 10 minutes, followed by 50 cycles of 95°C for 15 seconds and 60°C for 1 minute. Gene expression levels were normalized to the housekeeping gene *Gapdh*.

### Statistical Analysis

Each end point of interest (inflammation score, collagen area, hydroxyproline content per prostate weight, collagen synthesis measurement, quantitation of inflammatory cell subtypes and fibrocytes, collagen remodeling-associated gene expressions) was evaluated between saline instilled and *E. coli* infected animals at various time points. We employed a two-sample t-test or Wilcoxon rank-sum test to compare the group difference as appropriate. To explore the relationship between collagen area and inflammation score for each prostatic lobe, Spearman's rank correlation coefficient (ρ) was calculated. All analyses were conducted using SAS 9.2 (SAS Institute, Cary NC) software. A P-value<0.05 was considered statistically significant in two-tailed statistical tests.

## Supporting Information

Figure S1 **No significant difference in collagen content of the prostate was observed between saline-instilled and naïve controls.** Hydroxyproline content of prostate tissues from age-matched naïve C3H/HeOuJ male mice and saline-instilled C3H/HeOuJ mice 28 days post-instillation. n = 8 for naïve group, n = 9 for saline-instilled group. Data are presented as hydroxyproline (µg)/prostate weight (mg) ± SEM. Comparison of the hydroxyproline content between the two groups was performed by two-sample t-test. Hydroxyproline (HYP).
(PDF)

Figure S2 **Collagen subtype gene expressions in bacterial-induced prostatic inflammation.** qRT-PCR for *Col4a1, Col6a1,* and *Col6a*2 in the DLP from saline instilled and *E. coli* infected animals 7 and 28 days post-instillation. n = 4–7 per treatment per time point. Data are presented as mean gene expression ± SEM. Gene expression levels were normalized to the housekeeping gene *Gapdh.* Comparisons of the gene expressions between saline instilled and *E. coli* infected animals were performed by two-sample t-test. Post-Instillation Day (PID); Dorsolateral prostate (DLP).
(PDF)

Figure S3 **αSMA+VIM+ myofibroblast accumulation is not evident in bacterial-induced prostatic inflammation.** Immunohistochemical staining for αSMA (Green), vimentin (Red), and Hoechst (blue) in the saline instilled **(A)** and *E. coli* infected **(B)** prostates 7 days post-instillation. Scale bar 100 µm in panel A. Urogenital sinus obtained from an 18-day-old mouse embryo was used as a positive control for αSMA+VIM+ myofibroblasts (data not shown). Vimentin (VIM); α-smooth muscle actin (αSMA).
(PDF)

Table S1 **Primer Sequences (5′-3′) Used for RT-PCR Analysis.**
(DOC)
