# Supplementary figures and images for: Prostatic Inflammation Induces Fibrosis in a Mouse Model of Chronic Bacterial Infection
Source: PLoS One. 2014 Jun 20;9(6):e100770. doi: 10.1371/journal.pone.0100770 (PMC4065064; doi:10.1371/journal.pone.0100770)

# Hydroxyproline Content

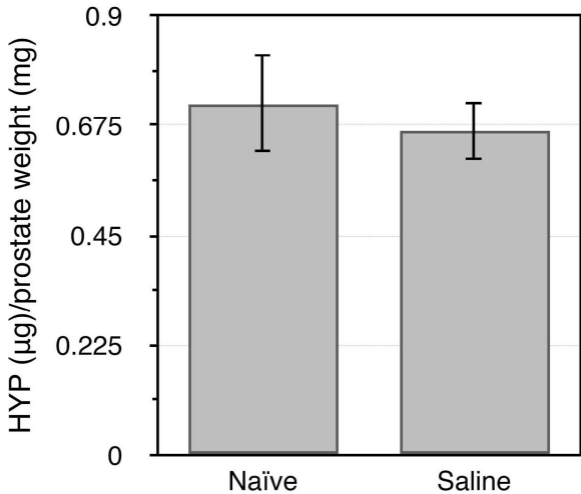

Supplement: Figure S1 — No significant difference in collagen content of the prostate was observed between saline-instilled and naïve controls. Hydroxyproline content of prostate tissues from age-matched naïve C3H/HeOuJ male mice and saline-instilled C3H/HeOuJ mice 28 days post-instillation. n = 8 for naïve group, n = 9 for saline-instilled group. Data are presented as hydroxyproline (µg)/prostate weight (mg) ± SEM. Comparison of the hydroxyproline content between the two groups was performed by two-sample t-test. Hydroxyproline (HYP). (PDF) [file pone.0100770.s001.pdf]

*Col4a1*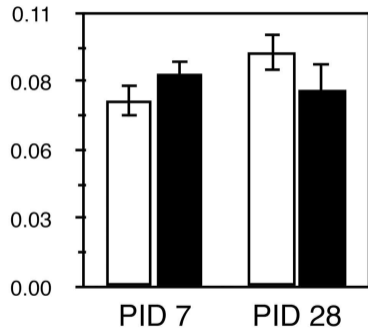*Col6a1*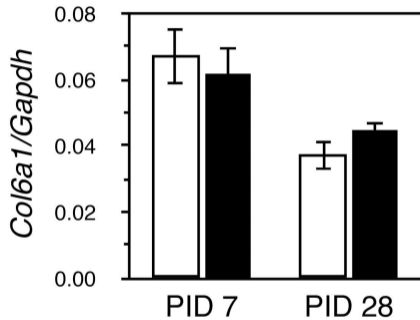*Col6a2*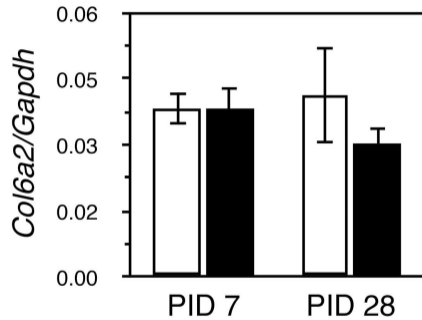

□ Saline DLP      ■ E. coli DLP

Supplement: Figure S2 — Collagen subtype gene expressions in bacterial-induced prostatic inflammation. qRT-PCR for Col4a1, Col6a1, and Col6a2 in the DLP from saline instilled and E. coli infected animals 7 and 28 days post-instillation. n = 4–7 per treatment per time point. Data are presented as mean gene expression ± SEM. Gene expression levels were normalized to the housekeeping gene Gapdh. Comparisons of the gene expressions between saline instilled and E. coli infected animals were performed by two-sample t-test. Post-Instillation Day (PID); Dorsolateral prostate (DLP). (PDF) [file pone.0100770.s002.pdf]

**Saline**

**E. coli**

**Hoechst +  $\alpha$ SMA + VIM**

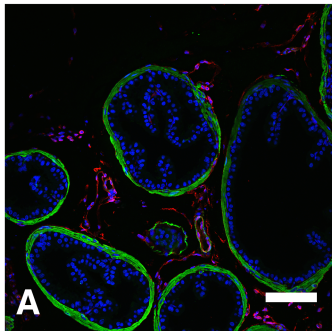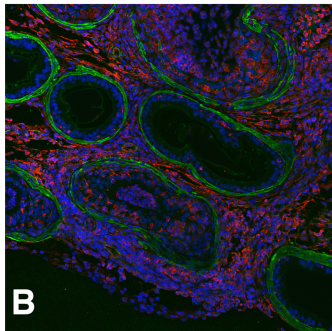

Supplement: Figure S3 — αSMA+VIM+ myofibroblast accumulation is not evident in bacterial-induced prostatic inflammation. Immunohistochemical staining for αSMA (Green), vimentin (Red), and Hoechst (blue) in the saline instilled (A) and E. coli infected (B) prostates 7 days post-instillation. Scale bar 100 µm in panel A. Urogenital sinus obtained from an 18-day-old mouse embryo was used as a positive control for αSMA+VIM+ myofibroblasts (data not shown). Vimentin (VIM); α-smooth muscle actin (αSMA). (PDF) [file pone.0100770.s003.pdf]
