# Supplementary material for: Prostatic Inflammation Induces Fibrosis in a Mouse Model of Chronic Bacterial Infection
Source: PLoS One. 2014 Jun 20;9(6):e100770. doi: 10.1371/journal.pone.0100770 (PMC4065064; doi:10.1371/journal.pone.0100770)
Supplement: Table S1 — Primer Sequences (5′-3′) Used for RT-PCR Analysis. (DOC) [file pone.0100770.s004.doc]

| **Table S1. Primer Sequences (5’-3’) Used for RT-PCR Analysis** | | |
| --- | --- | --- |
| **Gene** | **Forward Sequence** | **Reverse Sequence** |
| *Col1a1* | CCCCAAAAGCCAAAAATATG | TTTTGGTCACGTTCAGTTGG |
| *Col1a2* | ACACCCTGACACCTGTTGTG | ACAAGTGGTGCGAATGTTCA |
| *Col3a1* | CAACCAGTGCAAGTGACCAA | TGACCTGTATTGGGTGGTTG |
| *Mmp2* | TCCGCGTAAAGTATGGGAAC | ATCACTGCGACCAGTGTCTG |
| *Mmp7* | AGCTATGCAGCTCACCCTGT | GAGCCTGTTCCCACTGATGT |
| *Mmp9* | CATTCGCGTGGATAAGGAGT | TCACACGCCAGAAGAATTTG |
| *Mmp13* | AGTTGACAGGCTCCGAGAAA | GGCACTCCACATCTTGGTTT |
| *Timp3* | TTGGGTACCCTGGCTATCAG | TTGCTGATGCTCTTGTCTGG |
| *Lox* | TTCTTCTGCTGCGTGACAAC | AAACCAGCTTGGAACCAGTG |
| *Gapdh* | AGAACATCATCCCTGCATCC | CACATTGGGGGTAGGAACAC |
